# Antibacterial and Antiproliferative Activities of Plumericin, an Iridoid Isolated from *Momordica charantia* Vine

**DOI:** 10.1155/2015/823178

**Published:** 2015-04-07

**Authors:** Jutamas Saengsai, Sumonthip Kongtunjanphuk, Nuttawan Yoswatthana, Tanawan Kummalue, Weena Jiratchariyakul

**Affiliations:** ^1^Department of Pharmacognosy, Faculty of Pharmacy, Mahidol University, 447 Sri-Ayudhya Road, Rajthevi, Bangkok 10400, Thailand; ^2^Department of Biotechnology, Faculty of Applied Science, King Mongkut's University of Technology North Bangkok, Bangsue, Bangkok 10800, Thailand; ^3^Department of Chemical Engineering, Faculty of Engineering, Mahidol University, 25/25 Puttamonthon 4 Road, Salaya, Nakhon Pathom 73170, Thailand; ^4^Department of Clinical Pathology, Faculty of Medicine Siriraj Hospital, 2 Prannok Road, Bangkoknoi, Bangkok 10700, Thailand

## Abstract

Plumericin, an iridoid lactone, was isolated with relatively high yield from *Momordica charantia* vine using the supercritical fluid extraction (SFE) and the separation box (Sepbox) comprising dual combination of high-performance liquid chromatography and solid phase extraction. This compound showed antibacterial activity against *Enterococcus faecalis* and *Bacillus subtilis* with minimum inhibitory concentration (MIC) values better than cloxacillin. Plumericin potently inhibited proliferation of two leukemic cancer cell lines: they were acute and chronic leukemic cancer cell lines, NB4 and K562, with the effective doses (ED_50_) of 4.35 ± 0.21 and 5.58 ± 0.35 *μ*g/mL, respectively. In addition, the mechanism of growth inhibition in both cell lines was induced by apoptosis, together with G2/M arrest in K562 cells.

## 1. Introduction

*Momordica charantia* L., family Cucurbitaceae, commonly known as bitter gourd or bitter melon, is widely distributed in Asia and tropical Africa. All parts of the plant are bitter. It has been used as traditional medicine in Asia, South America, and Africa for a long time. Many publications report the therapeutic use of this plant [1–7].

The leaf extracts (water, ethanol, and methanol) of* M. charantia* showed the broad-spectrum antimicrobial activity [8].* In vitro* antimicrobial activity of leaf extract was seen against* Escherichia coli, Salmonella paratyphi, Shigella dysenteriae*, and* Streptomyces griseus* [9]. In phase II clinical study, the leaf extract showed the inhibition of* Mycobacterium tuberculosis* growth using the BACTEC 460 susceptibility test method [10]. All evidences supported that the aerial parts of* M. charantia* had potential as antibacterial activity; however, there is no report on the bioactive compound for this activity.

The preliminary studies (*in vitro* as well as* in vivo*) of* M. charantia* leaf and vine extracts and their purified compounds indicated the anticancer activity, whereas fruit and leaf extracts had chemopreventive effect on DMBA induced skin tumorigenesis, melanoma tumor, and cytogenicity [11]. The leaf methanol extract inhibited B16 (murine skin), HL-60 (human leukemia), MCF-7 (human breast), and HCT-8 (human colon) tumor cell lines with IC_50_ values less than 30 *μ*g/mL [12]. It also had the anti-invasive effect on a rat prostate cancer cell line (PLS10) and antimetastatic effect (*in vitro* and* in vivo*) [13]. Kuguacin J, isolated from* M. charantia* leaves, has been reported as inhibitor of P-glycoprotein (ABCB1) activity* in vitro* [14] and exerted a strong growth inhibitory effect on androgen-independent human prostate cancer (PC3) cell line* in vitro* [15]. Cucurbitane triterpenoids isolated from a leaf methanol extract showed potent inhibitory effects on skin-tumor promotion in an* in vivo* two-stage mouse skin carcinogenesis test, HL60 and SK-BR-3 cell lines [16]. The leaf Tris-HCl buffer extracts showed antihepatitis B virus activity and a mild cytotoxicity effect on the HepG2 cells [17]. It was also reported for antimigratory effects of human lung adenocarcinoma CL1 cells by reducing the expression and activation of Src and FAK to decrease the expression of downstream Akt, *β*-catenin, and MMPs [18].

In phytochemical study, the extraction and isolation processes usually take time between 3 weeks to several months. The technologies that can reduce time used for the extraction and isolation processes are preferable. Supercritical fluid extraction (SFE) is the advanced extraction technology applied in food and pharmaceutical and nutraceutical industries. Carbon dioxide is commonly used as solvent in SFE with the advantage over the conventional extraction in the absence of the residual solvent after the process. The solubility of chemical constituents in SFE process can be enhanced by the addition of cosolvent, such as ethyl acetate and ethanol [20]. SFE provides the extracts containing the target compounds in shorter time and minimizes the degradation of thermolabile components [19].

Sepbox or Separation box is a fully automated chromatographic instrument having dual combination of high-performance liquid chromatography (HPLC) and solid phase extraction (SPE) which permits the enrichment and isolation of highly diluted compounds. This instrument is used for the fractionation of plant extracts, prefractionation of extracts, and purification of the bioactive compounds. The recovery rate for both polar and nonpolar substances is usually above 90%. Sepbox produces high qualitative and reproducible results using an automated chromatographic system with two detectors (UV and ELSD detector). This is an efficient and fast separation and isolation technology [21, 22].

In this study,* M. charantia* vine was extracted with SFE and the target compound was fractionated and isolated with Sepbox. The extracts, the fractions, and the pure compound were tested for antibacterial activity. The antibacterial active fraction and the isolated compound were further studied for antiproliferative effect including cell cycle determination. The isolated compound was identified using spectroscopic methods including UV, FT-IR, MS, and NMR measurements.

## 2. Material and Methods

### 2.1. Plant Material

*Momordica charantia* vines were collected from Ayudhya province, Thailand, in January 2012. The plant was identified by the taxonomist from the Department of Forestry, Thailand, and the voucher number of the plant specimen was BKF. number 100085, SN 108121. The fresh vines were cleaned, cut into small pieces, dried in oven at 60°C for 8 h, and then ground to coarse powder for the extraction.

### 2.2. Extraction and Isolation

**Figure 1:**
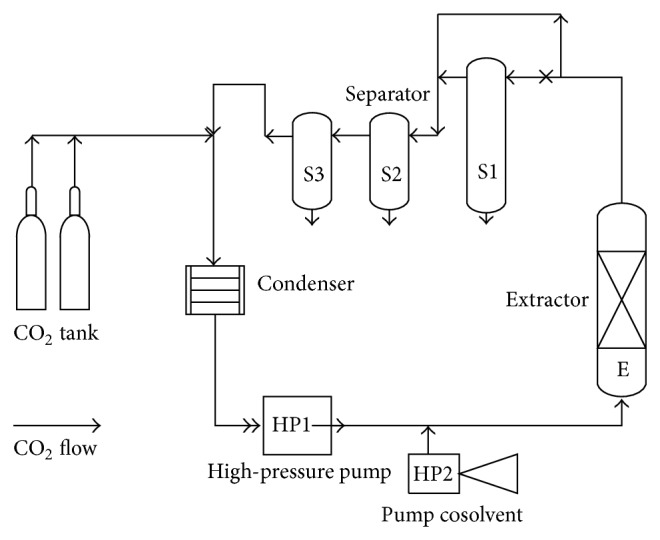
Schematic diagram of supercritical fluid extractor.

**Figure 2:**
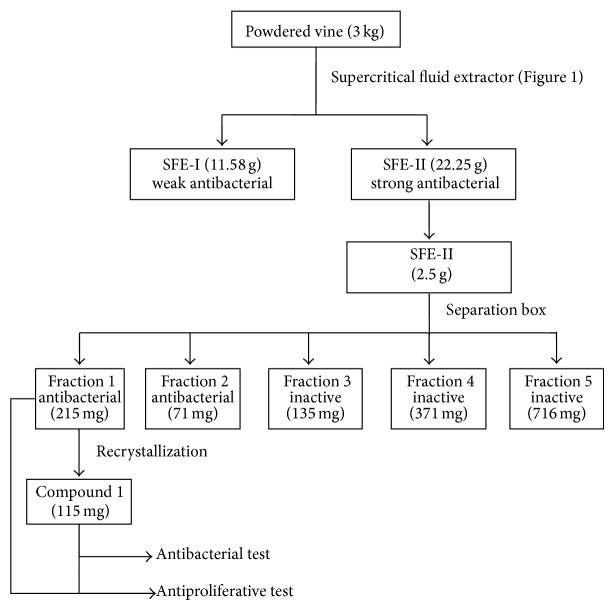
Bioassay-guided extraction and isolation.

The extractions were performed using Soxhlet apparatus and supercritical fluid extractor (SFE, Figure 1). The isolation was achieved with Separation box instrument. The extracts and the isolated compound were tested for antibacterial and antiproliferative effects as shown in Figure 2.

#### 2.2.1. Supercritical Fluid Extraction (SFE)

Supercritical fluid extraction (SFE) of pilot plant (Guangzhou Masson, China) belongs to Thai Traditional Medicine Development Center. The schematic diagram was shown in Figure 1. Three kg of* M. charantia* powder was loaded into the extraction thimble of the extractor column (E). Carbon dioxide was pumped through a high-pressure piston pump and cosolvent was pumped through a cosolvent syringe pump. The extraction thimble was installed in a temperature-controlled oven with coil of tube inside the oven which was used to preheat the CO_2_ and cosolvent before entering the extraction thimble. High-pressure pump, which was designed and operated for pressure up to 30 MPa, was used to pressurize CO_2_ for achieving supercritical state. The temperature and pressure in the extractor tanks were controlled and monitored by system controller.

After exiting the extractor, the extract (fluid) passed through the separators (S1, S2, and S3). S1 contained packing material for separating the natural pigments. S2 and S3 were empty tank with the regulated pressure and temperature. S2 received the nonvolatile substances and S3 for the volatile substances.

In this study, the different extraction parameters were optimized at temperature of 50°C, pressure of 25 MPa, and the CO_2_ flow rate of 5 L/min. Two solvents, ethyl acetate and ethanol, were used as cosolvents with the flow rate of 0.003 L/min. The pressures and temperatures of S2 and S3 were regulated at 9 MPa, 50°C and 5 MPa, 30°C, respectively.

#### 2.2.2. Soxhlet Apparatus

*M. charantia* coarse powder (100 g) was placed inside a thimble and loaded into the Soxhlet apparatus. This sample was extracted exhaustively with 2 L of ethanol for 8 h. The extract was filtered and evaporated to obtain the viscous extract.

#### 2.2.3. Separation Box

The isolation was carried out on Sepbox 2D (Sepiatec, Germany), which belonged to Thai Traditional Medicine Development Center. The instrument was equipped with a Smartline manager 5050 pump controller, a Smartline 1000 Hi-pressure pump (Knauer, Germany), a fraction collector, a UV detector (Labtech, USA), and a Sedex 80 LT-ELSD detector (Sedere, France) connected with an air pump. It contained one injection column, one main separation C_4_ column, six secondary separation columns, and eighteen trap columns.

2.5 g of SFE extract was weighed, dissolved in 375 mL ethyl acetate, and sonicated in ultrasonic bath for 15 min. The solution was centrifuged at 8,000 rpm for 10 min. The clear supernatant was filtered through a 0.45 *μ*m membrane filter. Reversed phase C_4_-bonded carrier (10 g) was added to the extract solution and thoroughly mixed using rotary evaporator (35°C, 60–90 rpm) to allow the extract solution coating on C_4_ adsorbent. The extract granule (approximately 12 g) was obtained.

One g of the granules (containing 0.8 g of C_4_ carrier and 0.2 g of extract) was transferred to injection column. The separation was carried out on a C_4_ main separation column and operated using a gradient solvent system of water-acetonitrile, started from 50% acetonitrile, linearly increased to 100% acetonitrile over 50 min, and then held for 12 min. The flow rate was maintained at 4.5 mL/min. Five trap columns were used to collect fractions by time. They were equilibrated with water at the flow rate of 6 mL/min for 5 min per each trap prior to separations. The first trap column started 2 min after the separation. Fractions 1–4 were accumulated in trap columns for 10 min and fraction 5 was collected for 20 min. The fractionation process was detected and monitored by UV and ELSD (evaporative light scattering detector) detectors. UV detection was set at a wavelength of 254 nm and the carrier gas of ELSD detector was set at 3.5 bar with the temperature of 50°C. After each separation process, all fractions were eluted from trap columns using methanol as an eluent with the flow rate of 6 min/mL, 10 min/trap and the solvent was removed by rotary evaporator.

### 2.3. Identification

The melting point of the isolated compound was measured on Electrothermal 9100 melting point apparatus (Electrothermal, UK). The UV spectra were recorded on Labtech UV2000 spectrophotometer (Labtech, USA). The IR spectrum was recorded on a Perkin Elmer FTIR 2000 spectrophotometer (Perkin Elmer, USA) using CHCl_3_ as a solvent, measured in cm^−1^. ESI-MS was recorded on a Bruker micrOTOF (Bruker, USA). ^1^H-NMR, ^13^C-NMR, DEPT 90, DEPT 135, and 2D-NMR spectra (COSY, HMQC, and HMBC) were measured on Bruker AV500D (Bruker, USA) in CDCl_3_.

### 2.4. Antibacterial Assay

Three Gram-negatives (*Escherichia coli* (ATCC 25922),* Pseudomonas aeruginosa* (ATCC 9027), and* Salmonella typhimurium* (CCM 5445)), and five gram-positives [*Staphylococcus aureus* (ATCC 25933),* Staphylococcus epidermidis* (ATCC 14990),* Enterococcus faecalis* (ATCC 29212),* Streptococcus mutans* (ATCC 25175), and* Bacillus subtilis* (ATCC 6633)] bacterial strains were used in the study.

One loop of the cultured bacteria was put into Erlenmeyer flask containing 200 mL nutrient broth (NB) and incubated in shaking incubator at 250 rpm, 37°C temperature for 24 h. After incubation, cell suspensions were diluted to 1 : 10 in NB and incubated at 37°C with 250 rpm shaking. The cell growths were studied using the optical density (OD) at 600 nm and detected every 60 min until the growth curves were obtained. The maximum growth rates were estimated from the log phase of the growth curves. The bacteria in the maximum growth rate were serially diluted to concentration range of 30–300 CFU/mL for determination of minimum inhibitory concentration (MIC).

The bioassay-guided fractionation was performed using disk diffusion test [23–26]. Bacterial suspensions were spread on Mueller-Hinton agar (MHA). Thirty microlitres of test sample solutions (concentration of 1 mg/mL) were dropped on 0.45 *μ*m sterile membrane filter (5 mm), used as antibiotic disc. Place the sample disc on the bacterial plates and incubated at 37°C for 24 h. The diameters of the inhibition zones were measured by using vernier caliper. The zones of inhibition were continuously observed for 5 days. The results of day 1 and day 5 were recorded.

The minimum inhibitory concentration (MIC) of the extracts, the fractions, and the isolated compound was obtained by broth dilution method [25–27]. Stock solutions of extracts and fractions were prepared at concentration of 5 mg/mL in dimethyl sulfoxide (DMSO). The concentration of isolated compound was prepared as 1 mg/mL in DMSO. From the preliminary experiment, antibiotics as kanamycin, ampicillin, amoxicillin, and cloxacillin were tested and only cloxacillin could kill all bacterial strains. For this reason, cloxacillin was used as positive control, and 10% DMSO in water was used as negative control.

The bacterial suspensions with concentration range of 30–300 CFU/mL (100 *μ*L) were added into 1.5 mL Eppendorf tubes, followed by 100 *μ*L of extracts, and then vortexed until the suspensions were obtained. The suspensions were spread on plate count agar (PCA) medium and incubated overnight at 37°C. For the MIC values, 100 *μ*L of the first suspensions was added to the next Eppendorf tubes and the procedure was repeated. Serial dilutions were carried out until the visible growth of bacteria was observed. The MIC values were read at the lowest concentration that produced invisible growth.

### 2.5. Antiproliferative Effect

#### 2.5.1. Cell Lines and Culture Conditions

Acute promyelocytic leukemia (NB4) and human chronic myeloid (K562) cell lines were maintained in RPMI 1640 media (GibThai, Thailand) supplemented with 10% fetal bovine serum (Stem Cell Technology, Canada) and 1% penicillin-streptomycin (GibThai, Thailand) in a humidified atmosphere with 5% CO_2_ [28].

Human hepatocellular carcinoma cell line (C3A, ATCC CRL-10741, USA) was maintained in MEM (GibThai, Thailand) containing 10% fetal bovine serum, 1% nonessential amino acid (NEAA, GibThai, Thailand), 1% sodium pyruvate (GibThai, Thailand), and 1% penicillin-streptomycin at 37°C in a humidified atmosphere with 5% CO_2_.

Human lung cancer (A549), human breast cancer (T47D), and normal human fibroblast (PromoCell, Germany) cell lines were maintained in DMEM media (GibThai, Thailand) supplemented with 10% fetal bovine serum and 1% penicillin-streptomycin at 37°C in a humidified atmosphere with 5% CO_2_ [29].

#### 2.5.2. Preparation of Test Samples

The test samples (fraction 1 (50 mg) and compound 1 (5 mg)) were weighed in Eppendorf tubes, 500 *μ*L of DMSO was added for fraction 1 and 100 *μ*L for compound 1, and then they were sonicated in ultrasonic bath until completely dissolved. The stock solution at concentration of 100,000 and 50,000 *μ*g/mL was obtained, respectively.

#### 2.5.3. Antiproliferative Assay

Antiproliferative assay of control (DMSO) and treated cells was evaluated by using the MTT (3-(4,5-dimethylthiazol-2-yl)-2,5-diphenyltetrazolium bromide) (Sigma, USA) assay in triplicate and three independent experiments [30, 31]. Human cancer cells at 1 × 10^4^ cells per well were seeded in a 96-well plate and incubated for 24 h before treating with the vinyl extract. Cells were treated with various final concentrations of extract fractions and isolated compounds at 0.1, 0.5, 1, 5, 10, 20, and 50 *μ*g/mL for 48 h. Fifty microlitres of MTT solution (1 mg/mL of PBS) was added to each well, followed by incubation for 4 h in a humidified atmosphere of 5% CO_2_ at 37°C. For NB4 and K562 cell lines, 100 *μ*L of SDS in 0.01 M HCl (Sigma) was added to each well, and then cells were incubated overnight in the incubator before measuring the OD on the next day. For other adherent cells, the growth medium was discarded from the wells and replaced with 100 *μ*L of DMSO to dissolve the formazan crystals. After 10 min of incubation at room temperature, the absorbance of the wells was read using an ELISA reader (Biotek Laboratories, USA) at a wavelength of 595 nm. Effective dose inhibiting 50% of cells was calculated as ED_50_ and formula of cell growth inhibition was as follows [32, 33]:

(1) $$\begin{array}{c} \text{Cell}\;\;\text{growth}\;\;\text{inhibition}\;\;\left(\%\right)=100-\frac{\text{sample}\;\;\text{O}.\text{D}.}{\text{control}\;\;\text{O}.\text{D}.}\times 100. \end{array}$$

### 2.6. Cell Cycle Determination

To evaluate the inhibitory effect of isolated compound, cell cycle analyses were investigated. Only two cancer cell lines, that is, NB4 and K562, were determined in these analyses because of the very strong inhibition. Both cancer cells were seeded in 6-well plates at the density of 1.5 × 10^6^ cells per well. Cells were treated with the isolated compound at concentrations of 0.5x ED_50_ and 1x ED_50_ with untreated cells as the control group. After 48 h, cell cycle studies were performed using CycleTEST PLUS DNA Reagent Kit (BD, Biosciences, USA) according to the manufacturer's protocol. All samples were analysed within 3 h by FACScalibur using CellQuest software.

## 3. Results and Discussion

Our preliminary study showed that the vine extract had antibacterial activity. The study in depth on the antibacterial compound was performed.

After 3 h of SFE extraction (Figure 1), the yield of S3 extract was very low (Table 1). It indicated the low content of volatile substances in* M. charantia* vine. For this reason, SFE extracts from the separator S2 (SFE-I, S2 and SFE-II, S2) were used in this study.

SFE with ethyl acetate cosolvent (SFE-I), SFE with ethanol cosolvent (SFE-II), and Soxhlet extracts were evaluated for their antibacterial activity (Table 2). Only SFE-II was selected for further investigation because it inhibited all bacterial strains.

**Figure 3:**
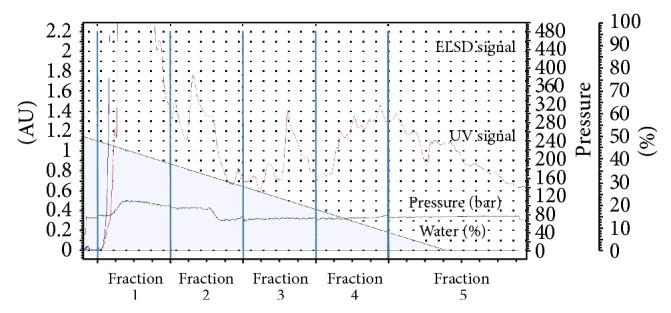
Fractionation chromatogram of SFE-II from Separation box.

SFE-II was chromatographed on injection column of Separation box. It was fractionated into 5 fractions using the gradient solvent system of water-acetonitrile (50%→100% acetonitrile) for 62 min. The chromatogram of the fractionation process (fractions 1–5) was shown in Figure 3. All fractions were tested for antibacterial activity and the zones of inhibition of each fraction were recorded (Table 3). Fraction 1 was further investigated for antiproliferative effect because it inhibited all microbial strains and the quantity obtained was three times higher than fraction 2. This fraction was washed with hot methanol to remove the dark brown pigment and then dissolved in chloroform and left for recrystallization. Compound 1, brown crystal with the melting point of 210-211°C, was obtained. Fraction 1 contained 53.5% of compound 1, SFE-II extract 4.6% of compound 1, and the powdered vine 0.03% of compound 1 (Figure 2). Compound 1 on day 1 showed the antibacterial activity against all bacterial strains (Table 3). The MIC values of compound 1 against* Enterococcus faecalis* and* Bacillus subtilis* were better than cloxacillin (Table 4).

There were several reports on antiproliferative effect of* M. charantia*. We thus performed the antiproliferative effect of fraction 1 and compound 1. The results showed that fraction 1 and compound 1 had strong inhibitory effect against leukemic (NB4, K562) and moderate effect on liver cancer (C3A) cell lines. Fraction 1 was very interesting because it could inhibit C3A better than compound 1 and produced less cytotoxicity to normal human fibroblast cell line (HF) (Table 5). Fraction 1 could not inhibit lung (A549) and breast (T47D) cancer cell lines. However, compound 1 could inhibit T47D at the modest level but it also produced cytotoxicity to HF.

**Figure 4:**
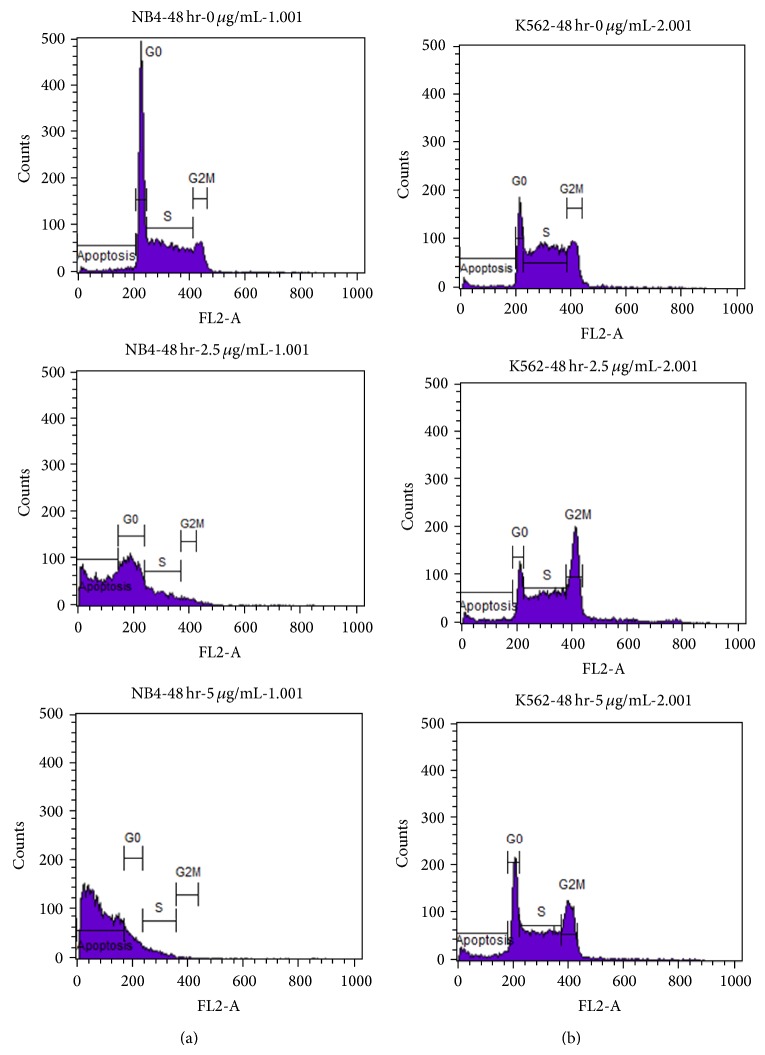
Dot plot analyses on cell cycle distribution of NB4 and K562 cell lines ((a) NB4, (b) K562). Details of data were shown in Table 6.

To evaluate the mechanism of the strong inhibitory action by compound 1 on NB4 and K562, cell cycle analyses of these two leukemic cell lines were conducted. Interestingly, the apoptosis of NB4 was exhibited as the prominent mechanism, whereas the apoptosis and also G2/M arrest were demonstrated in K562 cells, as shown in Figure 4 and Table 6.

**Figure 5:**
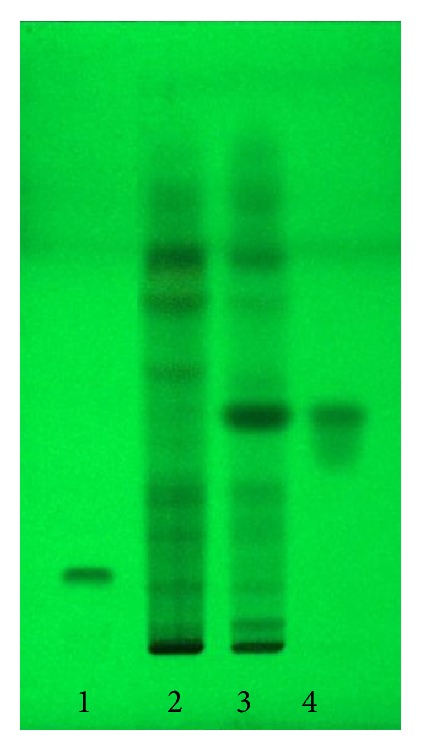
Thin-layer chromatogram of cucurbitacin B as a reference compound (1), SFE-II (2), fraction 1 (3), and compound 1 (4); adsorbent: silica gel GF_254_, solvent system: hexane-ethyl acetate (6 : 4), and detector: under UV 254 nm.

Thin-layer chromatogram of SFE-II, fraction 1, and compound 1 was shown in Figure 5. The chemical structure of compound 1 was characterized as follows.

Compound 1 produced the *hR*_*f*_ value of 41 on the precoated silica gel aluminum sheet with the solvent system of hexane-ethyl acetate (6 : 4) and the detection as dark spot under UV 254 nm. The UV spectrum showed the maximum absorption in methanol at 230 nm. The IR spectrum indicated the aliphatic carbonyl group (C=O) stretching at 1756 cm^−1^ and C=O attached to the ring at 1705 cm^−1^. The C-H stretching of the skeleton showed around 3083 cm^−1^ and C=C stretchings at 1684, 1646, 1617, and 1438 cm^−1^. The ESI mass spectrum displayed the molecular ion peaks at 313.07 [M + Na]^+^ and 603.13 [2M + Na]^+^, suggesting a molecular formula of C_15_H_14_O_6_ with the molecular weight of 290.

The chemical structure of compound 1 was elucidated using ^1^H and ^13^C-NMR data, which coincided with plumericin [34] as shown in Table 7.

**Figure 6:**
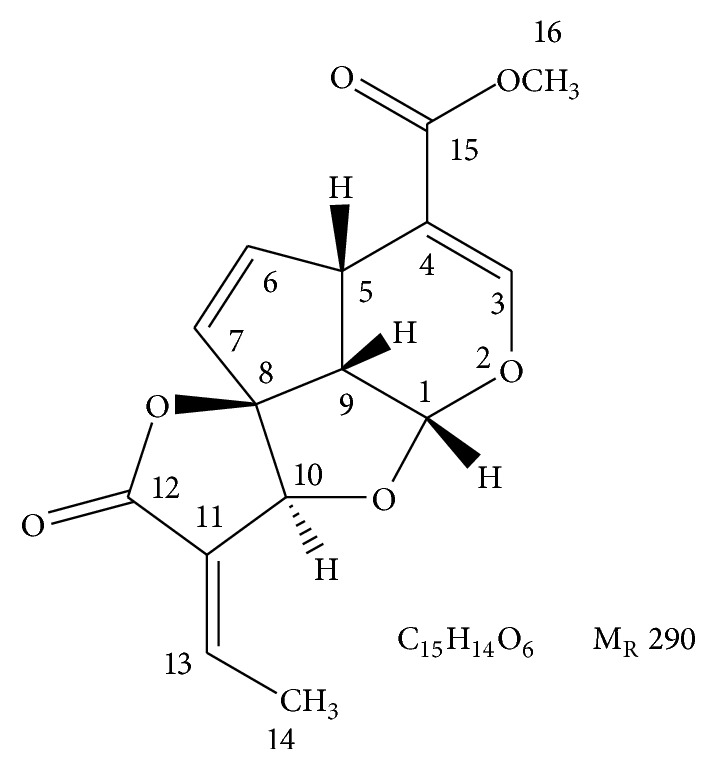
Structure of plumericin.

From the spectroscopic evidences shown in Table 7, the structure of compound 1 was elucidated as an iridoid lactone, plumericin (Figure 6). This compound has been previously found in apocynaceous plants [34–40]. This is the first report on plumericin in cucurbitaceous plant,* Momordica charantia*.

Compound 1: plumericin, C_15_H_14_O_6_, recrystallized in chloroform as brown plates; mp. 210-211°C; UV (*λ*_max⁡_ in methanol): 230 nm; IR (*ν*_CHCl_3__): 3083, 2951, 1756, 1705, 1646, 1438, 1081, 1023, 867, 798, 763 cm^−1^; ESI-MS *m*/*z*: 603.13 (2M + Na), 313.07 (M + Na); ^1^H-NMR (400 MHz, CDCl_3_) *δ*: 7.42 (1H,* s*, H-3), 7.15 (1H,* dq*, *J* = 7.2, 1.4 Hz, H-13), 6.03 (1H,* dd*, *J* = 5.4, 2.2 Hz, H-6), 5.63 (1H,* dd*, *J* = 5.4, 2.1 Hz, H-7), 5.55 (1H,* d*, *J* = 5.9 Hz, H-1), 5.09 (1H,* br.s*, H-10), 4.00 (1H,* ddd*, *J* = 9.5, 2.2, 2.1 Hz, H-5), 3.75 (3H,* s*, H-16), 3.42 (1H,* dd*, *J* = 9.5, 5.9 Hz, H-9), 2.07 (3H,* d*, *J* = 7.2 Hz, H-14); ^13^C-NMR (100 MHz, CDCl_3_) *δ*: 168.42 (C-12), 166.90 (C-15), 152.94 (C-3), 145.54 (C-13), 141.31 (C-6), 127.68 (C-11), 126.61 (C-7), 109.58 (C-4), 104.83 (C-8), 102.49 (C-1), 80.53 (C-10), 53.91 (C-9), 51.90 (C-16), 38.63 (C-5), 16.33 (C-4).

The previous biological activities of plumericin included antibacterial, antifungal, antileukemic, anticancer, cytotoxic, and anti-inflammatory activities [35–45]. Our study discovered that plumericin was the major constituent of* M. charantia* vine and had high antibacterial and antiproliferative effects.

## 4. Conclusion

Antibacterial compound, plumericin, was isolated for the first time from* M. charantia* vine using the advanced extraction and separation technologies. Supercritical fluid extraction provided a rapid extraction of a specific group of compounds while the Separation box achieved a rapid isolation of the pure compounds. Plumericin showed the antibacterial activity against 8 pathogenic bacterial strains, especially* E. faecalis* and* B. subtilis* with the MIC values better than cloxacillin, the positive control. It also had the high antiproliferative effect against leukemic (NB4 and K562), breast cancer (T47D) cell lines, and moderate effect against liver cancer cell line (C3A). We also discovered that fraction 1 was promising drug material for further clinical study on liver cancer.

## Supplementary Material

The spectroscopic data of compound 1 (ESIMS, DEPT, HMQC, HMBC and COSY) were supplemented. ESIMS indicated the molecular mass of compound 1 as 2M+Na and M+Na with m/z 603.13 and 313.07, respectively. DEPT identified the carbon types. HMQC illustrated the direct attached proton to carbon (1JC-H) and HMBC the vicinal protons (3JC-H). The H, H-COSY showed the vicinal protons (3JH-H). The diagram of separation box (sepbox) was also supplemented.

## Figures and Tables

**Table 1:** Yields of *M. charantia* vine extracts from Soxhlet apparatus and SFE.

Extract (weight of dry sample)	Weight (g)	% yield (g/100 g)
Soxhlet (100 g)	8.55	8.55
SFE-I, S2 (3,000 g)	11.58	0.39
SFE-I, S3 (3,000 g)	2.13	0.07
SFE-II, S2 (3,000 g)	22.25	0.74
SFE-II, S3 (3,000 g)	1.21	0.04

Supercritical fluid extract with ethyl acetate cosolvent (SFE-I), Supercritical fluid extract with ethanol cosolvent (SFE-II).

**Table 2:** Zones of inhibition of *M. charantia* extracts.

Sample	Day	Zones of inhibition (mm)							
		*Ec. *	*Pa. *	*Sa. *	*Se. *	*Ef. *	*St. *	*Sm. *	*Bs. *
Soxhlet	1	5.22	5.76	7.15	5.68	5.75	5.42	—	—
	5	—	—	—	—	—	—	—	—

SFE-I	1	6.08	5.65	—	5.74	—	—	—	—
	5	—	—	—	—	—	—	—	—

SFE-II	1	6.90	8.14	6.94	7.70	7.20	7.45	6.58	6.94
	5	5.62	6.34	5.78	6.45	—	—	—	—

*Escherichia coli* (*Ec.*), *Pseudomonas aeruginosa* (*Pa.*), *Staphylococcus aureus* (*Sa.*), *Staphylococcus epidermidis* (*Se.*), *Enterococcus faecalis* (*Ef.*), *Salmonella typhimurium* (*St.*), *Streptococcus mutans* (*Sm.*), and *Bacillus subtilis* (*Bs.*).

**Table 3:** Zones of inhibition produced by fractions from Separation box.

Sample	Day	Zones of inhibition (mm)							
		*Ec. *	*Pa. *	*Sa. *	*Se. *	*Ef. *	*St. *	*Sm. *	*Bs. *
Fraction 1 (5 mg/mL)	1	15.23	16.50	15.66	17.12	15.83	15.50	14.13	15.43
	5	13.00	11.00	10.55	13.25	15.70	12.14	6.96	12.80

Fraction 2 (5 mg/mL)	1	10.87	14.21	10.12	16.22	—	8.75	8.22	7.95
	5	9.53	12.66	9.78	13.42	—	7.80	7.10	7.22

Fraction 3 (5 mg/mL)	1	—	—	—	—	—	—	—	—
	5	—	—	—	—	—	—	—	—

Fraction 4 (5 mg/mL)	1	—	—	—	—	—	—	—	—
	5	—	—	—	—	—	—	—	—

Fraction 5 (5 mg/mL)	1	—	—	—	—	—	—	—	—
	5	—	—	—	—	—	—	—	—

Compound 1 (1 mg/mL)	1	6.51	7.84	6.12	6.72	8.64	7.66	7.22	7.52
	5	4.90	6.75	5.45	5.42	—	—	6.36	—

Cloxacillin (1 mg/mL)	1	12.24	11.25	6.47	7.21	7.64	9.12	16.50	7.22
	5	7.22	6.40	—	—	—	—	—	—

*Escherichia coli* (*Ec.*), *Pseudomonas aeruginosa* (*Pa.*), *Staphylococcus aureus* (*Sa.*), *Staphylococcus epidermidis* (*Se.*), *Enterococcus faecalis* (*Ef.*), *Salmonella typhimurium* (*St.*), *Streptococcus mutans* (*Sm.*), and *Bacillus subtilis* (*Bs.*).

**Table 4:** Minimum inhibitory concentrations (MIC) of SFE-II, compound 1, and positive control.

Sample	Minimum inhibitory concentration (*μ*g/mL)							
	*Ec. *	*Pa. *	*Sa. *	*Se. *	*Ef. *	*St. *	*Sm. *	*Bs. *
SFE-II	1000	1000	1000	1000	**1000**	1000	1000	**1000**
Compound 1	250	125	500	250	**250**	250	250	**125**
Cloxacillin	125	62.50	250	125	**1000**	125	15.62	**500**

*Escherichia coli* (*Ec.*), *Pseudomonas aeruginosa* (*Pa.*), *Staphylococcus aureus* (*Sa.*), *Staphylococcus epidermidis* (*Se.*), *Enterococcus faecalis* (*Ef.*), *Salmonella typhimurium* (*St.*), *Streptococcus mutans* (*Sm.*), and *Bacillus subtilis* (*Bs.*).

**Table 5:** ED_50_ of fraction 1 and compound 1 on six cell lines detected by MTT assay.

Sample	ED_50_ (*μ*g/mL) (mean ± SD)					
	NB4	K562	C3A	A549	T47D	HF
Fraction 1	5.00 ± 0.07	27.92 ± 0.18	9.93 ± 0.32	>50^*^	>50^*^	26.78 ± 1.38
Compound 1	4.35 ± 0.21	5.58 ± 0.35	25.21 ± 0.74	51.35 ± 11.94	10.55 ± 1.02	0.93 ± 1.18

^∗^Inactive.

**Table 6:** Cell cycle determination of NB4 and K562 cells showed that, at 48 h, the apoptosis significantly occurred when compared with the control group. Moreover, G2/M arrest was also detected in K562 cells.

NB4	Concentration (*μ*g/mL)	Apoptosis	G0	S	G2M
48 hrs	0	4.44	36.54	46.43	11.91
	2.5 (0.5ED_50_)	40.13	40	15.92	3.19
	5 (ED_50_)	73.46	16.22	8.96	0.89

K562	Concentration (*μ*g/mL)	Apoptosis	G0	S	G2M

48 hrs	0	4.15	16.01	58.39	18.5
	2.5 (0.5ED_50_)	5.27	11.98	44.25	31.72
	5 (ED_50_)	9.08	22.94	41.98	23.1

**Table 7:** ^1^H and ^13^C-NMR spectroscopic data of compound 1 in CDCl_3_.

C	*δ* ^13^C	*δ* ^∗^^13^C [19]	HMQC	HMBC
			*δ* ^1^H (*J*)	
1	102.49 D	102.8	5.55 *d* (*5.9*)	H-5, H-9
3	152.94 D	153.0	7.42 *s *	H-5
4	109.58 S	109.5		H-3, H-5
5	38.63 D	38.8	4.00 *ddd* (*9.5, 2.2, 2.1*)	H-3, H-6, H-7, H-9
6	141.31 D	141.0	6.03 *dd* (*5.4, 2.2*)	H-5, H-7, H-9
7	126.61 D	127.2	5.63 *dd* (*5.4, 2.1*)	H-5, H-6, H-10
8	104.83 S	105.0		H-1, H-6, H-7, H-9
9	53.91 D	53.9	3.42 *dd* (*9.5, 5.9*)	H-1, H-5, H-6, H-7
10	80.53 D	80.5	5.09 b*s *	H-13, H-14
11	127.68 S	128.3		H-14
12	168.42 S	168.4		H-10, H-13, H-14
13	145.54 D	144.6	7.15 *dq* (*7.2, 1.4*)	H-10, H-14
14	16.33 Q	15.8	2.07 *d* (*7.2*)	H-13
15	166.90 S	166.7		H-3, H-5
16	51.90 Q	51.4	3.75 *s *	

^∗13^C-NMR literature values.
