# Supplementary material for: Antibacterial and Antiproliferative Activities of Plumericin, an Iridoid Isolated from Momordica charantia Vine
Source: Evid Based Complement Alternat Med. 2015 Apr 7;2015:823178. doi: 10.1155/2015/823178 (PMC4405293; doi:10.1155/2015/823178)
Supplement: Supplementary file 1 — The spectroscopic data of compound 1 (ESIMS, DEPT, HMQC, HMBC and COSY) were supplemented. ESIMS indicated the molecular mass of compound 1 as 2M+Na and M+Na with m/z 603.13 and 313.07, respectively. DEPT identified the carbon types. HMQC illustrated the direct attached proton to carbon (1JC-H) and HMBC the vicinal protons (3JC-H). The H, H-COSY showed the vicinal protons (3JH-H). The diagram of separation box (sepbox) was also supplemented. [file 823178.f1.pdf]

Appendix : Supporting information

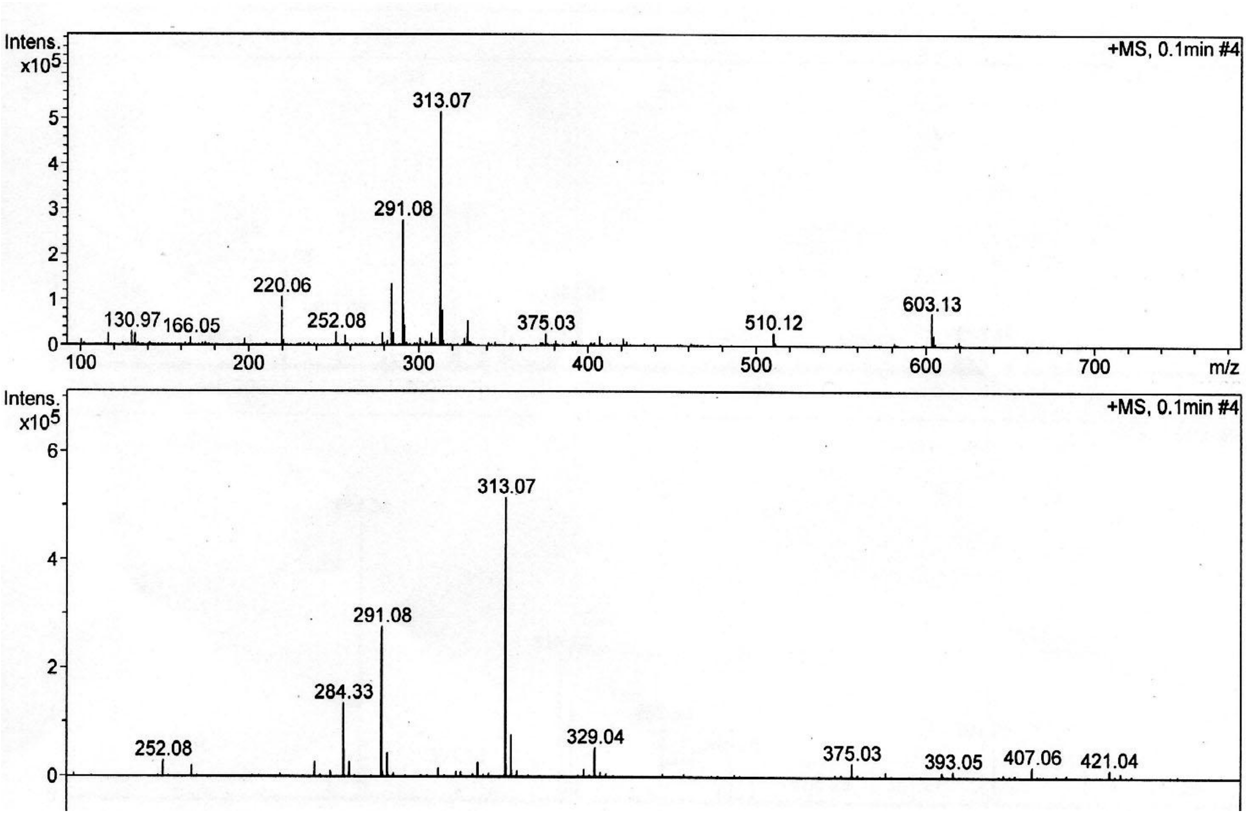

FIGURE A-1: ESI-MS spectrum of compound 1.

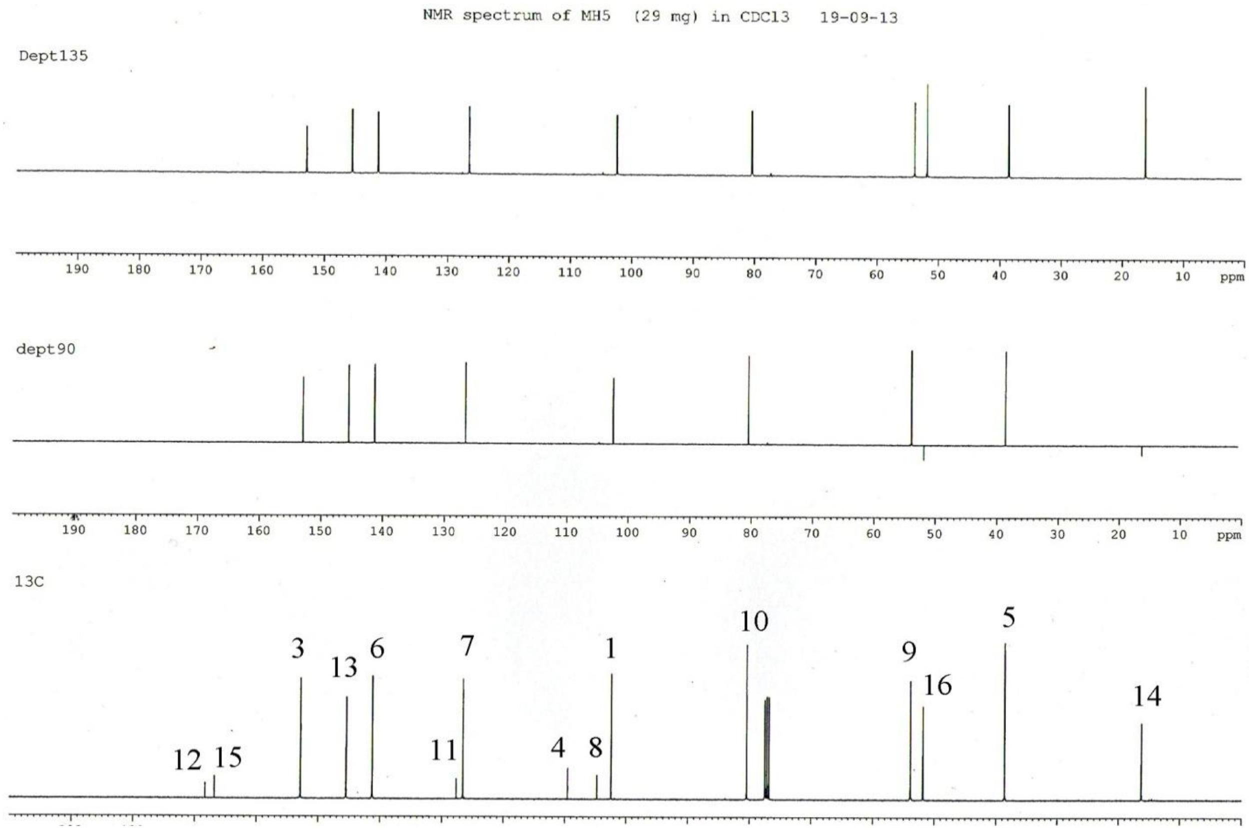

FIGURE A-2: The DEPT 90 and DEPT 135 spectrum of isolated compound 1.

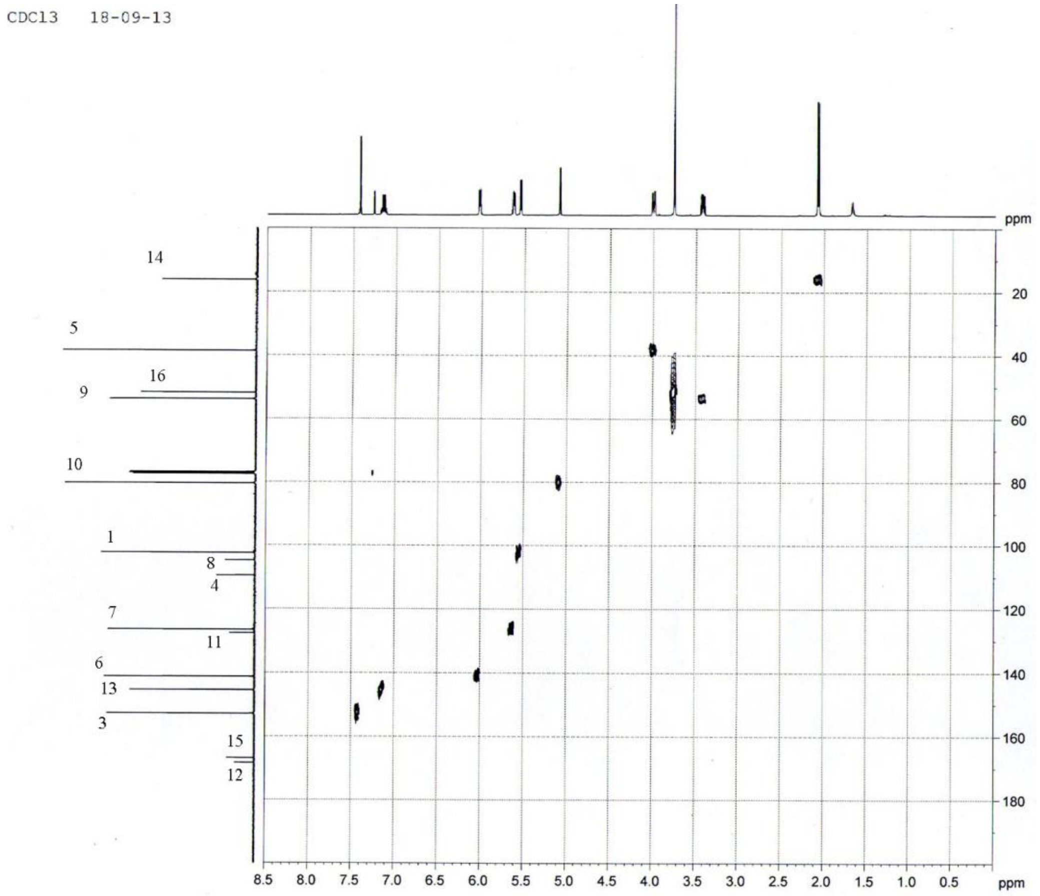

FIGURE A-3: HMQC spectrum of compound 1 in $CDCl_3$

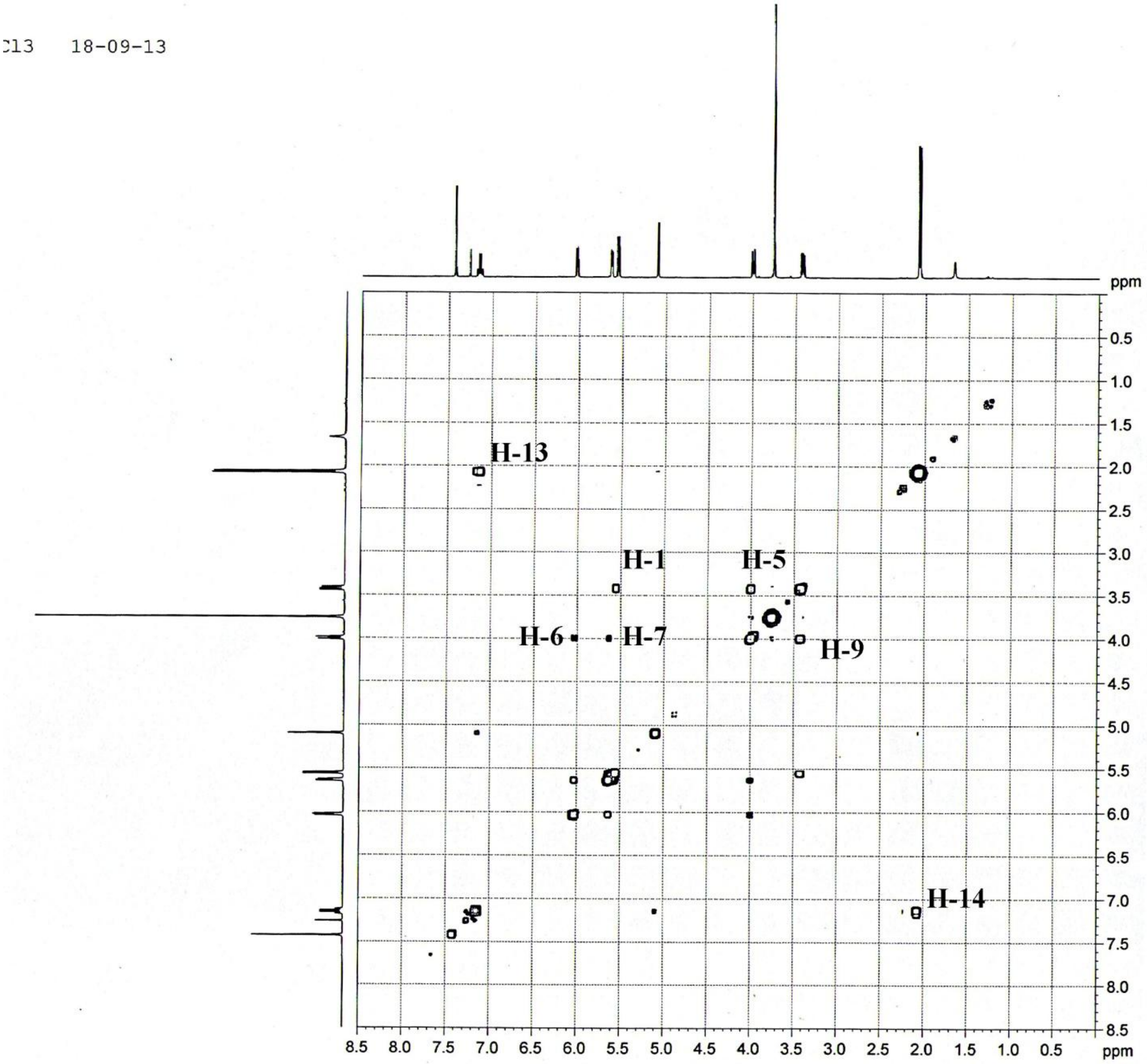

FIGURE A-4: H-H COSY spectrum of compound 1 in $CDCl_3$

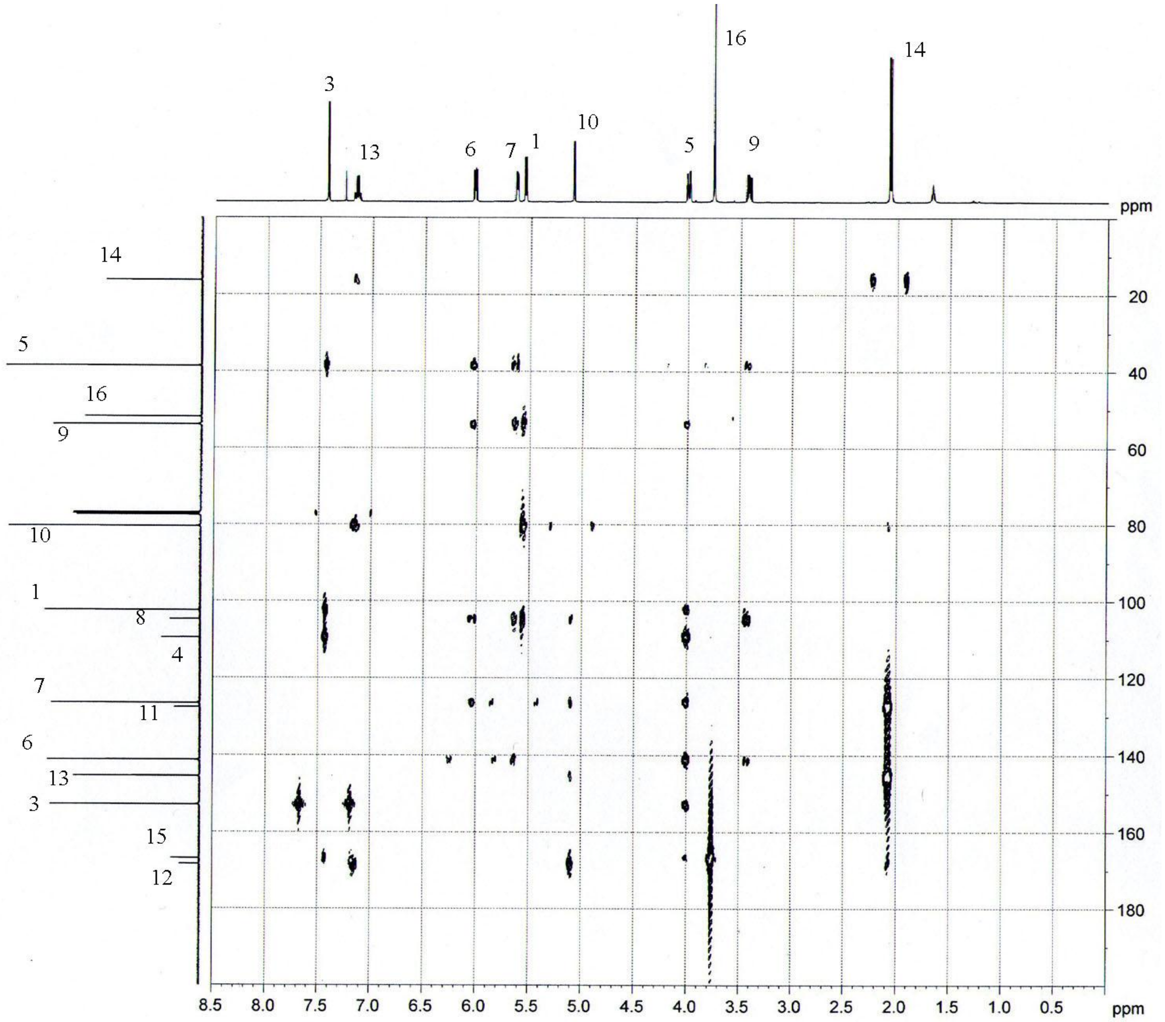

FIGURE A-5: HMBC experiment of compound 1.

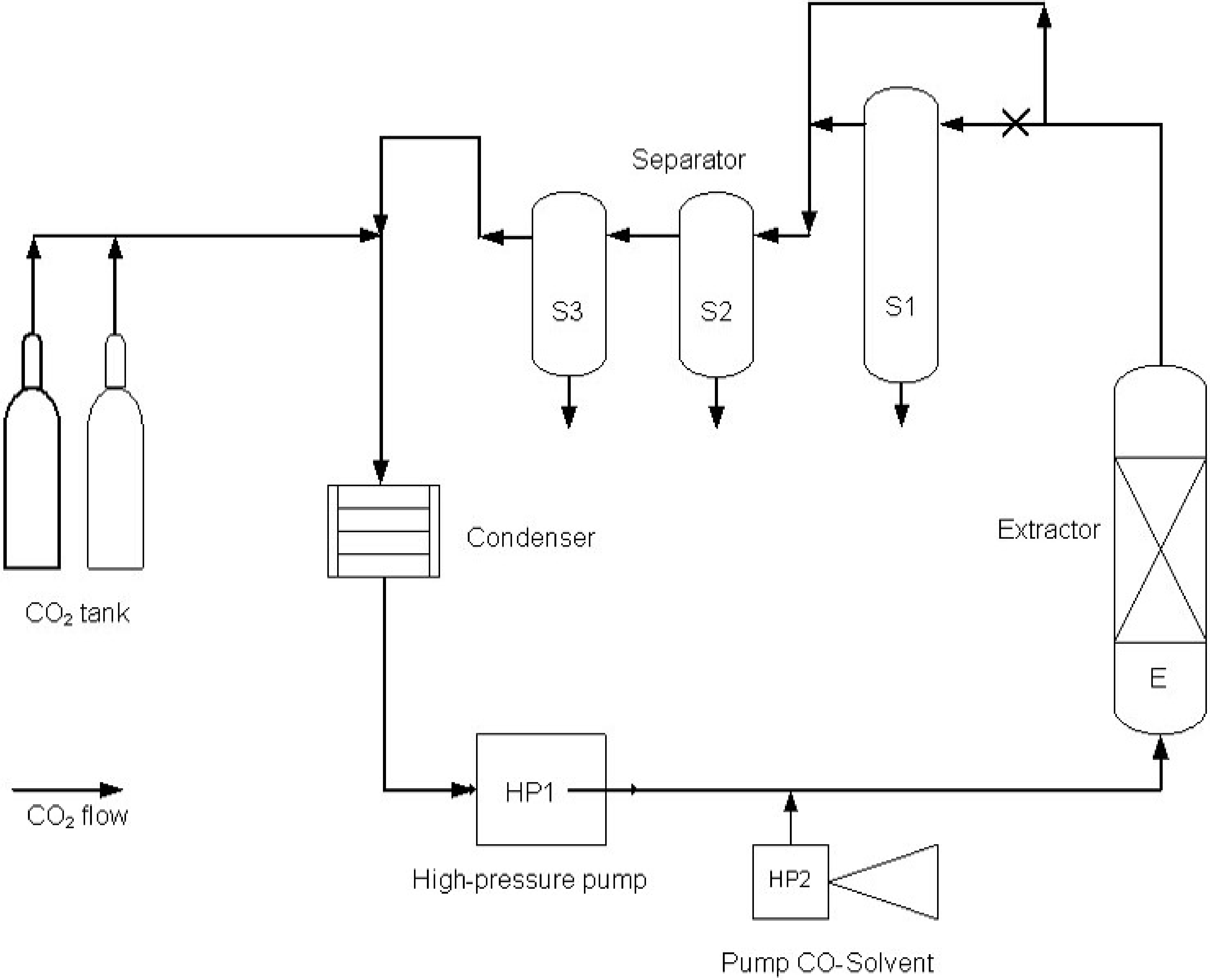

Separator
S3
S2
S1
Condenser
Extractor
E
CO2 tank
CO2 flow
HP1
High-pressure pump
HP2
Pump CO-Solvent

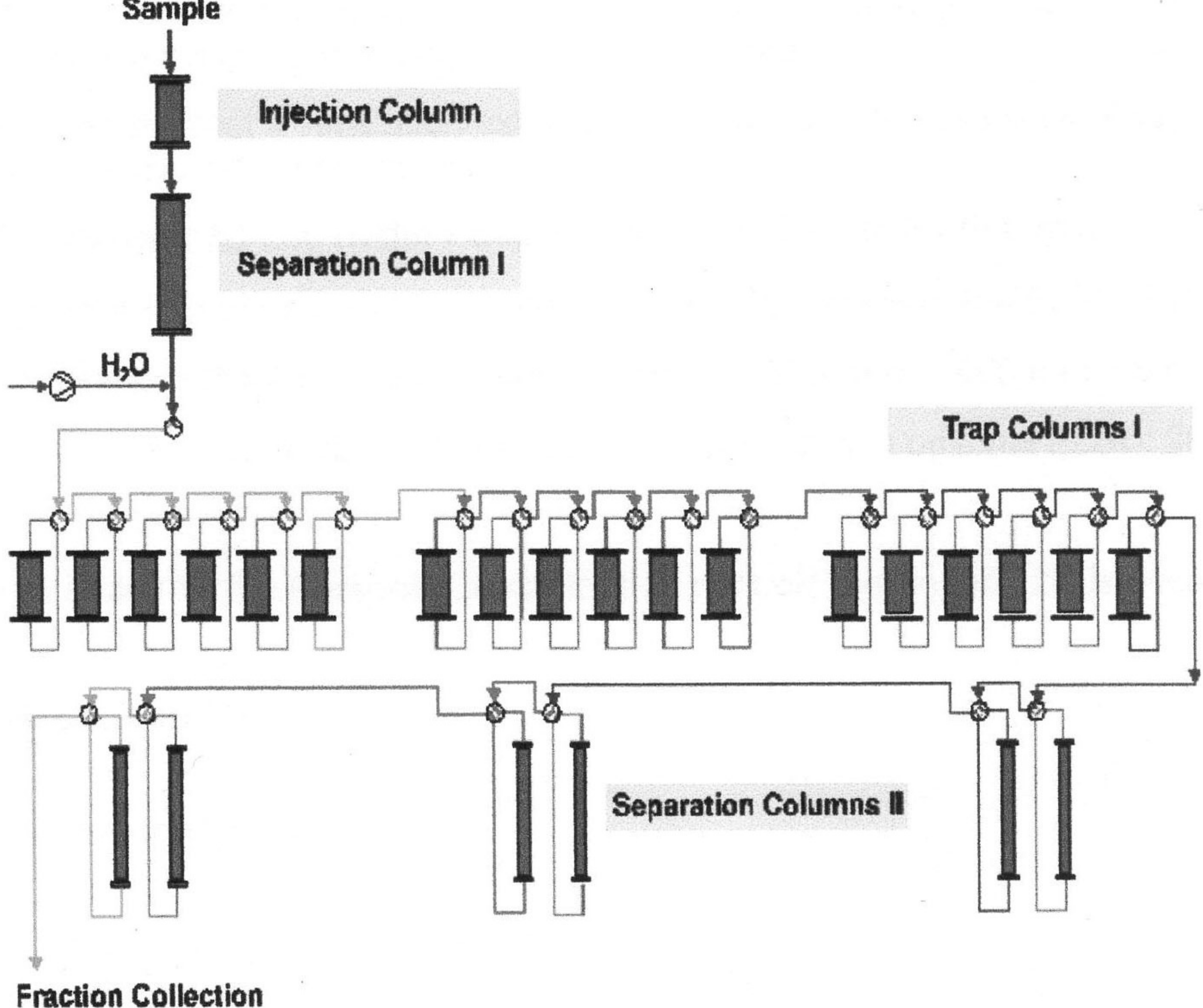
Sample
Injection Column
Separation Column I
H_2O
Trap Columns I
Separation Columns II
Fraction Collection

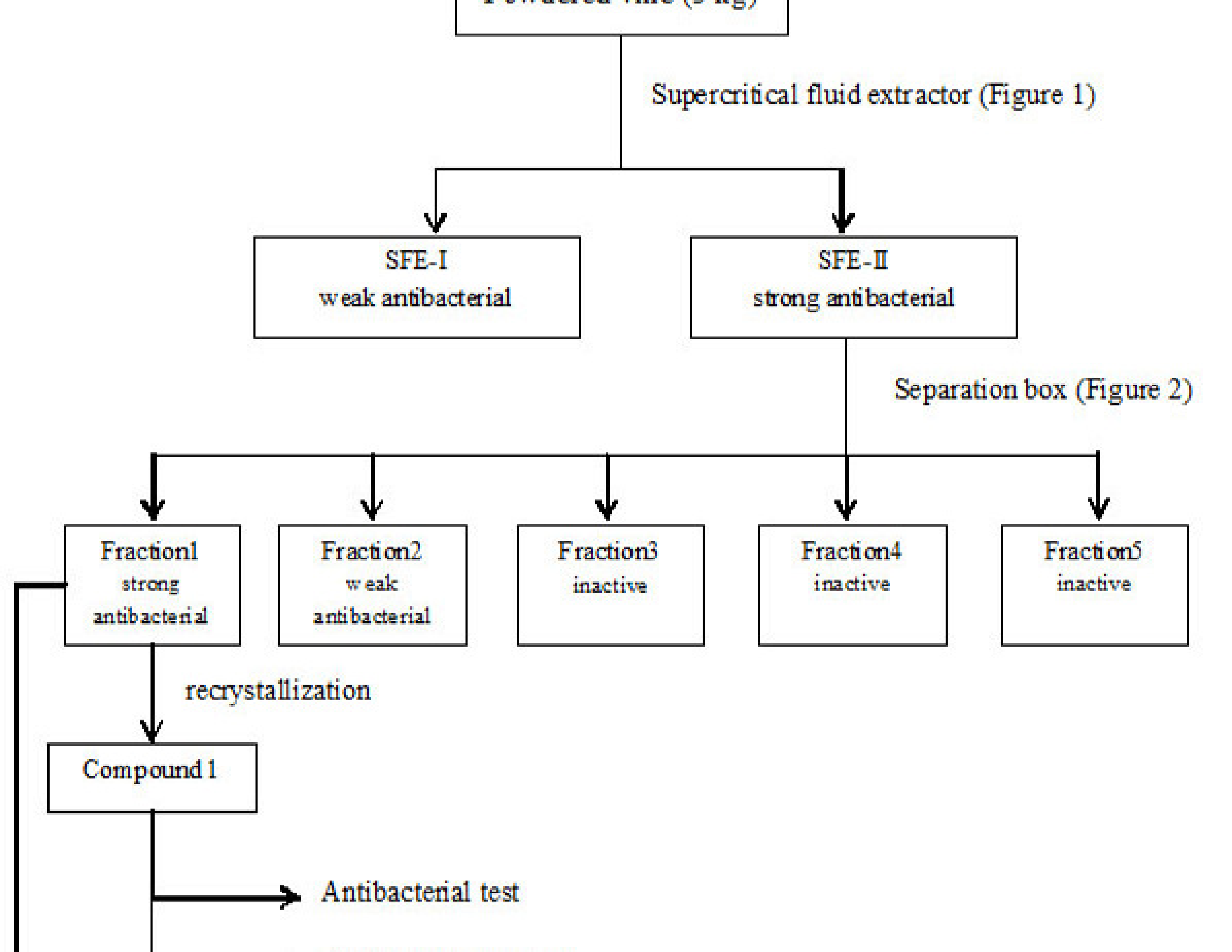
Supercritical fluid extractor (Figure 1)
SFE-I
weak antibacterial
SFE-II
strong antibacterial
Separation box (Figure 2)
Fraction1
strong
antibacterial
Fraction2
weak
antibacterial
Fraction3
inactive
Fraction4
inactive
Fraction5
inactive
recrystallization
Compound 1
Antibacterial test

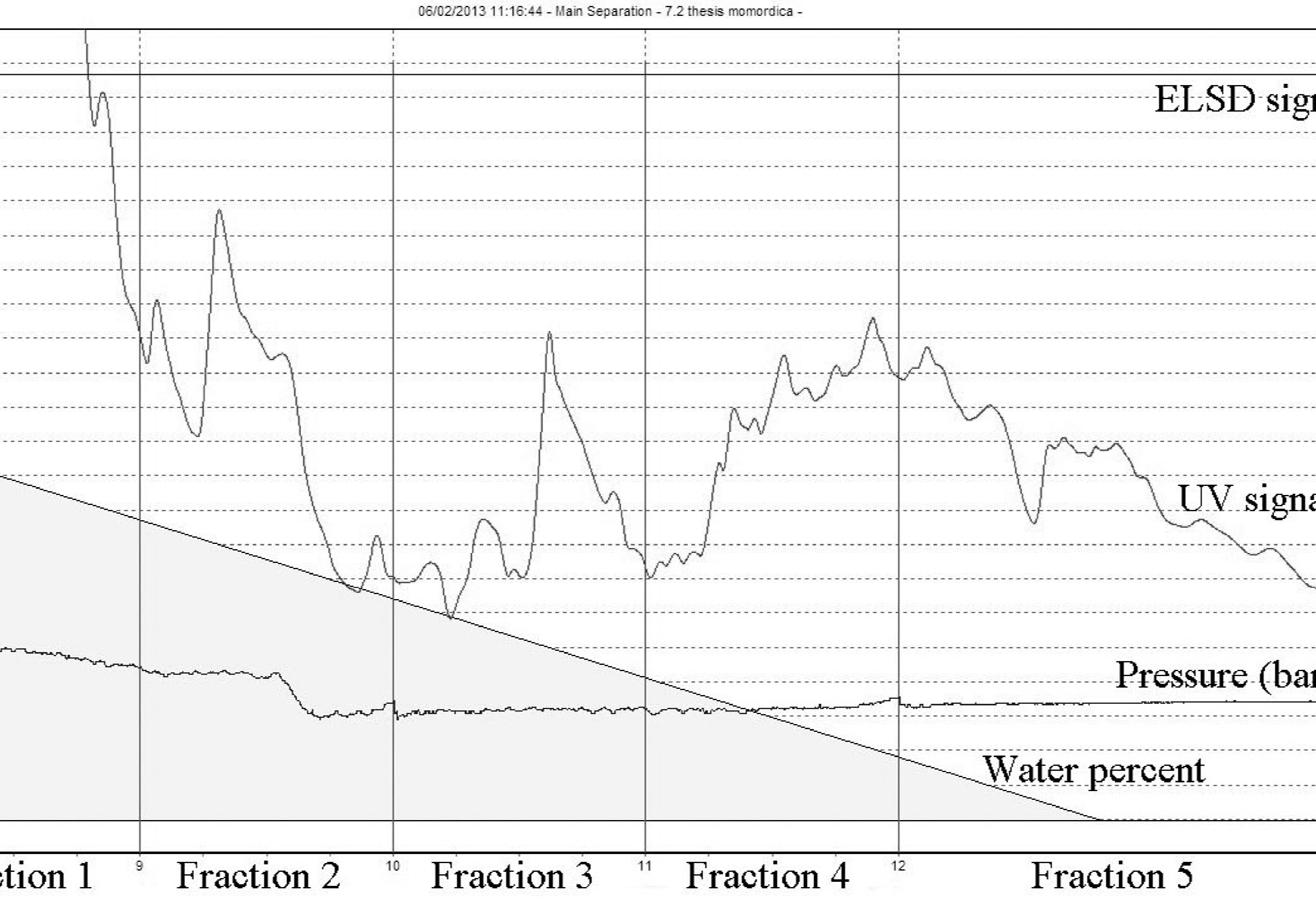

06/02/2013 11:16:44 - Main Separation - 7.2 thesis momordica -
ELSD sig
UV signa
Pressure (ba
Water percent
9
10
11
12
ction 1
Fraction 2
Fraction 3
Fraction 4
Fraction 5

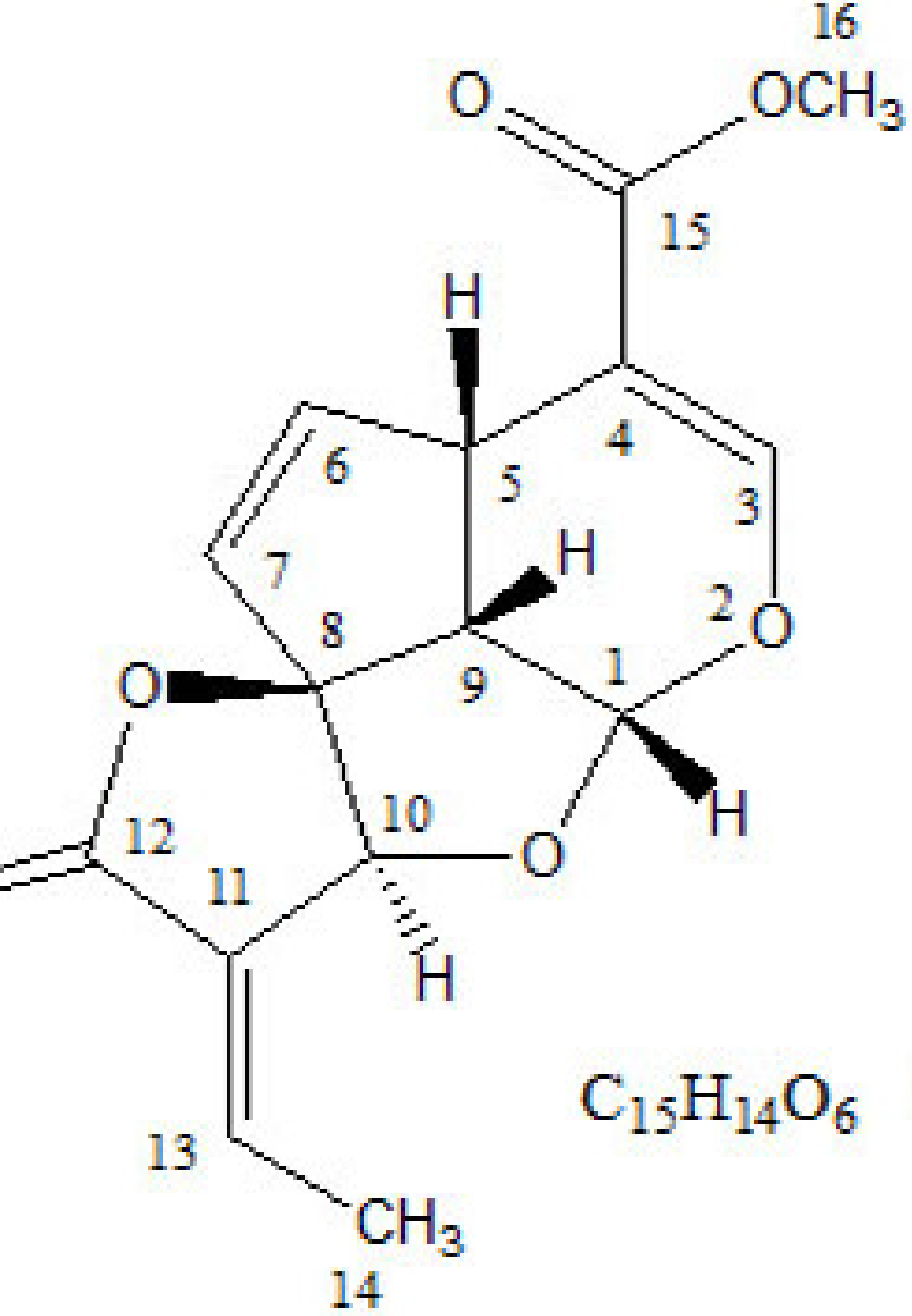

$C_{15}H_{14}O_6$ M_R
